# PP94 Using Plain Language to Empower Patient Expertise and Break Down Barriers for Equitable Participation in Health Technology Assessment

**DOI:** 10.1017/S0266462325103115

**Published:** 2025-12-29

**Authors:** Antonella Cardone, Jose Diaz, Martin Coombes

## Abstract

**Introduction:**

Patient organizations giving input to health technology assessment (HTA) often receive little information on the treatment being assessed. To promote equitable assessment and enhance trust and transparency, patient organizations need clear and accessible information. The use of plain language summaries as part of the HTA process was evaluated to examine how they can help break down barriers and empower patient involvement in HTA.

**Methods:**

The experience of piloting the Summary Information for Patient groups template in the HTA process in England was assessed using surveys, interviews, an advisory board, and a short-life working group. These qualitative data were evaluated by the HTAi Patient and Citizen Involvement in HTA Interest Group, including the issues and challenges, lessons learned, and recommendations for future adaptation to support patient involvement.

**Results:**

The pilot tests reported positive feedback on the Summaries by patient organizations, regardless of their level of HTA experience. The Summaries reduced preparation time and increased confidence in providing input. Other stakeholders also appreciated them. In a public consultation, 80 percent of respondents agreed or strongly agreed that “manufacturers should provide a ‘Summary of Information for Patients’ with their evidence submission.” However, HTA bodies highlighted the need to consider additional resources, timelines for incorporating Summaries into existing processes, and the need to review completed templates to mitigate bias. Feedback was analyzed and eight recommendations were proposed for consideration by HTA bodies and other stakeholders.

**Conclusions:**

Pilot tests have indicated a role for plain language summary information to support patient organization input and involvement in the HTA process, although there are issues and challenges that warrant further discussion. Nonetheless, recommendations can help establish best practice for stakeholders wanting to adapt the international Summary Information template and implement the approach to achieve more transparent and equitable assessments.

